# The Pathophysiological Roles of Regulatory T Cells in the Early Phase of Systemic Sclerosis

**DOI:** 10.3389/fimmu.2022.900638

**Published:** 2022-05-24

**Authors:** Satomi Kobayashi, Yasuo Nagafuchi, Hirofumi Shoda, Keishi Fujio

**Affiliations:** ^1^ Department of Allergy and Rheumatology, Graduate School of Medicine, The University of Tokyo, Bunkyo-ku, Japan; ^2^ Department of Medicine and Rheumatology, Tokyo Metropolitan Geriatric Hospital, Itabashi-ku, Japan; ^3^ Department of Functional Genomics and Immunological Diseases, Graduate School of Medicine, The University of Tokyo, Bunkyo-ku, Japan

**Keywords:** systemic sclerosis, the early disease phase, immune cells, regulatory T cells, transcriptome analysis

## Abstract

Systemic sclerosis (SSc) is an autoimmune disease that is characterized by vascular damage and fibrosis. Both clinical manifestations and immunological disturbances are diverse according to the disease duration. Particularly, changes in immunological processes are prominent in the early phase of SSc. The orchestration of several subsets of immune cells promotes autoimmune responses and inflammation, and eventually stimulates pro-fibrotic processes. Many reports have indicated that CD4^+^ T cells play pivotal roles in pathogenesis in the early phase of SSc. In particular, the pathogenic roles of regulatory T (Treg) cells have been investigated. Although the results were controversial, recent reports suggested an increase of Treg cells in the early phase of SSc patients. Treg cells secrete transforming growth factor-β (TGF-β), which promotes myofibroblast activation and fibrosis. In addition, the dysfunction of Treg cells in the early phase of SSc was reported, which results in the development of autoimmunity and inflammation. Notably, Treg cells have the plasticity to convert to T-helper17 (Th17) cells under pro-inflammatory conditions. Th17 cells secrete IL-17A, which could also promote myofibroblast transformation and fibrosis and contributes to vasculopathy, although the issue is still controversial. Our recent transcriptomic comparison between the early and late phases of SSc revealed a clear difference of gene expression patterns only in Treg cells. The gene signature of an activated Treg cell subpopulation was expanded in the early phase of SSc and the oxidative phosphorylation pathway was enhanced, which can promote Th17 differentiation. And this result was accompanied by the increase in Th17 cells frequency. Therefore, an imbalance between Treg and Th17 cells could also have an important role in the pathogenesis of the early phase of SSc. In this review, we outlined the roles of Treg cells in the early phase of SSc, summarizing the data of both human and mouse models. The contributions of Treg cells to autoimmunity, vasculopathy, and fibrosis were revealed, based on the dysfunction and imbalance of Treg cells. We also referred to the potential development in treatment strategies in SSc.

## Introduction

Systemic sclerosis (SSc) is a connective tissue disease which is characterized by the combination of immunological disturbances, impaired peripheral circulation, and fibrotic features, such as skin and lung fibrosis (1). Advanced fibrosis causes lethal organ dysfunction, such as heart and respiratory failure. Various approaches have been taken for treatment, including immunosuppressive reagents, vasodilation, and antifibrotic therapy (2, 3). These approaches have gradually improved the survival rate of SSc patients. Despite the success of these approaches, the 5-year-survival of diffuse cutaneous SSc, which involves systemic organs, remains relatively low at 85.5%. Therefore, SSc is an intractable disease with a high lethality rate compared to other connective tissue diseases (4).

SSc patients present with various clinical manifestations depending on their disease stages. For example, Raynaud’s phenomenon, suggesting a peripheral circulation disorder and microangiopathy, is a relatively early clinical manifestation of SSc (1). The definition of “the early disease phase” varies among reports. Typically, less than five years at longest (5, 6), and less than three years of disease duration represents the early phase, especially in diffuse cutaneous SSc (7, 8). A majority of SSc patients experience peripheral circulation disorders in the early phase of their disease courses. In particular, Raynaud’s phenomenon (RP) and nailfold capillary abnormalities are characteristic features of peripheral circulation disorders in the early phase of SSc (1, 9, 10), and are caused by vascular damage and dysfunction. Nailfold videocapillaroscopy (NVC) abnormality is another feature of peripheral circulation disorders and is easily observed in the very early phase of SSc. Abnormalities of nailfold capillaries, such as enlargement, bleeding, and stenosis, suggest vascular dysfunction and damage. These features are associated with a vasculopathy that is characterized by endothelial dysfunction and fibrotic proliferation (11). To diagnose very early SSc, predictors of developing definite SSc have been proposed. An international multicenter study on the very early diagnosis of SSc (VEDOSS) determined that disease-specific autoantibody and puffy fingers were independent parameters predicting the development of definite SSc. They also showed that puffy fingers have a positive predictive value (12). Bellando-Randone et al. additionally reported that the presence of one or two VEDOSS criteria (systemic sclerosis-specific autoantibodies, nailfold capillaroscopy abnormalities, and puffy fingers) in patients with Raynaud’s phenomenon confers a progressively higher risk for SSc over time (13).

The dysregulated activation of the immune system is observed from the early stage of the disease prior to fibrosis (14, 15). Several immune cell subsets, such as T cells, B cells, and macrophages, play pivotal roles in the pathogenesis of SSc. In recent years, the importance of CD4^+^ T cells, particularly regulatory T (Treg) cells, in the pathophysiology of SSc has emerged as an important player both in immune disorders and in the fibrotic pathogenesis of SSc. This paper focuses on the immunological features in the early phase of SSc and outlines the possible role of Treg cells in the pathophysiology of the early phase of SSc.

## Immunological Features of SSc

The main pathological features of the early phase of SSc are microvascular dysfunction and autoimmunity (16, 17).

Even though the precise mechanisms remain unknown, exposure to environmental risk factors, such as toxins, irritants, and viral infections, would trigger endothelial and epithelial damage (18, 19). These stimulants induce both innate and adaptive immune system activation, and inflammation. Indeed, the tissue infiltration of immune cells, predominantly around blood vessels, is prominent in the early phase of SSc. Although these immune cells mainly consist of CD4^+^ dominant T cells and macrophages (20), the orchestration of many types of immune cells promotes tissue inflammation, autoimmune responses, tissue damage, and eventually fibrosis.

Monocyte/macrophage subsets play crucial roles in innate immunity, tissue repair, and fibrosis (21). In a previous report, we performed bulk and single-cell RNA-sequencing (scRNA-seq) of peripheral blood immune cells from SSc and identified a SSc-related inflammatory gene module and a cluster of monocytes, similar to lung-infiltrating FCN1^hi^ monocytes expressing *IL1B*. This study included SSc patients with relatively long disease durations, therefore we estimated that monocyte/macrophage subsets were important for SSc pathogenesis during the disease course (22). On the other hand, monocyte/macrophage features that are prominent in the early disease phase of SSc were reported many years ago. York et al. reported that the expression of Siglec-1, an IFN-regulated marker, in circulating monocytes from early SSc and the number of Siglec-1^+^ cells in skin samples from early SSc were increased compared with healthy controls (HC) (23). Recently, Slaug et al. reported that M1 and M2 macrophage signatures were higher in early SSc than in late SSc (8).

Dendritic cells (DCs) are broadly classified as myeloid DC (mDC) and plasmacytoid DC (pDC). pDC secrete not only IFN but also cytokines and chemokines including CXCL4, which is an early SSc marker (24).

B cells have variable immune functions including the production of antibodies, antigen presentation, and cytokine production. Therefore, much research has focused on the role of B cells in SSc pathogenesis. Genome-wide association study (GWAS) identified several B cell-related genes, such as *BLK*, *CSK*, and *PTPN22*, as SSc susceptibility genes (25). Sato et al. reported that transgenic mice that overexpressed CD19 generated spontaneous autoantibodies, and that the CD19 density on blood B cells from SSc patients was significantly higher compared with HC. They implied that gene expression changes in B cells contributed to autoimmunity in SSc (26). Studies using SSc tissue samples, including lung and skin samples, have also been published. As for histopathological studies, Lafyatis et al. showed that the number of CD20^+^ cells was higher in the pulmonary tissues from patients with SSc-associated interstitial lung disease compared to controls (27). Bosselo et al. reported that the number of CD20^+^ cells was higher in clinically involved skin than in uninvolved skin, and there was a greater number of CD20^+^ cells in patients with early SSc compared with patients with long-standing disease (28). As described above, many types of immune cells play important roles in the pathogenesis of SSc. We intensively discuss the roles of CD4^+^ T cells in a later section.

## Genetics and Immunology in SSc

Genetic studies suggested the primary role of the immune system in the pathogenesis of SSc. A GWAS identified risk genes that are associated with innate and acquired immunity. For exaple, *IRF5*, one of the transcription factors involved in interferon (IFN) signaling, was the first interferon regulatory factor (IRF) gene reported to be associated with SSc (29). *TNFAIP3* encodes a ubiquitin-editing enzyme involved in the TNF-mediated immune response, and one SSc-associated genetic variant of *TNFAIP3* was strongly associated with the silica-induced profibrotic response of fibroblasts (30). *PTPN22* encodes the protein tyrosine phosphatase lymphoid tyrosine phosphatase, which is a critical gatekeeper of T-cell receptor (TCR) signaling. The *PTPN22* R620W polymorphism influences SSc genetic susceptibility (31). Notably, autoimmune diseases, such as systemic lupus erythematosus (SLE) and rheumatoid arthritis, share the same immune-related risk genes (32, 33). In addition, genes associated with fibrosis and vascular homeostasis were also identified as risk genes of SSc. For example, DDX6 is a regulator of vascular endothelial growth factor (34, 35). In this way, genetic analysis suggested that the pathogenesis of SSc is characterized by the combination of immune system dysregulation, vascular homeostasis, and fibrotic processes.

As described above, many immune cell subsets contribute to the pathogenesis of SSc, especially in the early disease phase. However, López-Isac et al. evaluated cell type-specific enrichment of SSc risk loci in representative epigenetic marks and reported that SSc-associated variants displayed significant enrichment in a histone mark of active genes in primary T cells and Natural Killer T cells (34). Moreover, in SSc, tissue-infiltrating T cells are predominantly CD4^+^ T cells, which express activation markers and show oligoclonal expansion (36). Taking these past reports into account, we focused on the role of CD4^+^ T cells in the immune system in early SSc.

## CD4^+^ T Cells in Early SSc

Many reports on CD4^+^ T cells in early SSc were published in this decade (7, 8, 37–53). Using skin samples from early SSc, Maehara et al. reported that microscopy and quantitative image analysis showed prominent infiltration of CD4^+^ cytotoxic T cells and an accumulation of apoptotic cells in the skin (37). The authors speculated that cytotoxic T cells might induce the apoptotic death of endothelial cells and contribute to vasculopathy in SSc. Skaug et al. revealed that immune cells (including CD4^+^ T cells) and fibroblast signatures positively correlated with the skin thickness and progression rate in early SSc by using transcriptomic analysis of the skin samples (8, 38). They also performed immunohistochemical staining and showed that most early diffuse cutaneous SSc patients with high baseline T cell and macrophage numbers had declines in these numbers at follow-up (8). Zhou et al. reported that quantitative reverse transcription PCR (RT-qPCR), immunohistochemistry (IHC), and Western blotting analysis showed the overexpression of T-helper17 (Th17)-related cytokines, such as IL-17A, in early SSc skin lesions. They reported that protein expression of IL-17A was positively correlated with the modified Rodnan skin score of SSc (39).

Studies with peripheral blood have yielded controversial results. Many reports have examined Treg cells. For example, Shah et al. reported that fluorescence-activated cell sorting (FACS) analysis of peripheral blood showed reductions in activated total T cells, CD4^+^ T cells, and FOXP3^+^CD25^+^ Treg cells, and a greater bias towards an IL4^+^ T helper 2 (Th2)/T cytotoxic 2 (Tc2) phenotype based on the ratio of Th2/Th1 CD4^+^ and Tc2/Tc1 CD8^+^ T cells (41). Kataoka *et al.* reported that FACS analysis of peripheral blood showed that the proportion of Foxp3^+^ cells among the total CD4^+^ T cells decreased in patients with either early- or late-stage SSc compared with HC (45). The authors implied that the lower expression level of Runx1 mRNA in purified Tregs in SSc was related to the frequency of Tregs in SSc. Conversely, several reports on early SSc revealed an increased proportion of Treg or Treg subpopulations (42–44), and some reports also showed an increased proportion of Th17 cells (42, 44).

The published reports on Treg cell and Th17 subsets of early SSc were listed in **Table 1** (7, 39, 40, 42–46, 54–59). The majority of the reports indicated that the frequencies of Treg cells in the blood or the skin were increased in early SSc in comparison with those in late SSc or HC (40, 42, 43). In addition, some reports showed the decreased regulatory capacity in Treg from SSc (43, 58). In the case of Th17 cells, a number of reports suggested the frequencies of Th17 or the levels of Th17-related cytokines were higher in the blood or the skin from early SSc (7, 40, 42, 44, 59), even though some articles reported that those were comparable between early and late SSc (57).

**Table 1:** The reports on Treg cell and Th17 subsets of early SSc.

Authors (reference)	Tissue	Results about CD4^+^ T cell subsets
Kobayashi, et al. (42)	blood	In FACS analysis, increases of Th17/CD4^+^ T cells in early SSc were found compared with late SSc (p < 0.001) and HC (p < 0.05). In RNA-seq, Treg genes were differentially expressed in early SSc compared with late SSc (False Discovery Rate < 0.05). Increases of “activated subpopulation” in Treg, calculated by deconvolution were found in early SSc compared with late SSc (p < 0.05) and HC (p < 0.05), and that was associated with OXPHOS pathway and TCR signaling.
Fox, et al. (7)	blood	In FACS analysis, the expansion of Th17 cells was shown in early dcSSc (p < 0.034). The number of Treg cells was similar in early dcSSc and control groups.
Kubo, et al. (54)	blood	According to the FACS data, SSc patients were grouped into three groups; Tfh-dominant, Treg-dominant, and fewer abnormalities group). The disease duration was comparable among the groups.
D’Amico, et al. (55)	SNP genotyping data	Using SNP genotyping data, the occurrence of FOXP3 rs2294020 in female patients was associated with decreased time to progression from early to definite SSc (allelic model: HR = 1.43; CI = 1.03-1.99; p = 0.03; dominant model: HR = 1.54; CI = 1.04-2.28; p = 0.03).
Krasimirova, et al. (46)	blood	Increases in TGF-β1 levels in the serum were found in SSc in the early stage against SSc in the late stage by ELISA (30.03 ± 4.59 ng/mL vs 13.08 ± 4.50 ng/mL, P = 0.017).
Ugor, et al. (43)	blood	In FACS analysis, CD4^+^CD25^+^Foxp3^+^CD127^-^ Treg cells were significantly elevated in patients with early dcSSc (p < 0.05) and in patients with anti-Scl-70/RNA-Pol-III autoantibody positivity (p < 0.05) and with lung fibrosis (p < 0.05). A significantly lower proportion of IL-10 producing Tregs was found in SSc (p < 0.05) compared to HC. Increased CD62L^+^ Treg cells were present in SSc compared to HC (p < 0.05). A decreased frequency of TGF-β producing CD62L^+^ Tregs was found in SSc compared to HC (p < 0.05).
Almanzar, et al. (44)	blood	In FACS analysis, the proportions of circulating peripheral IL-17^-^producing CCR6^+^ Th cells and FoxP3^+^ Tregs were increased in patients with dcSSc. In a multiple regression model (R^2^ = 0.995; p = 0.015) performed in dcSSc, higher IL-17 production by CD4^+^CCR6^+^ was significantly influenced by lower chronological age (p = 0.004), shorter disease duration (p = 0.033), organ involvement (p = 0.012), and increased inflammation parameters (ESR, p = 0.021; CRP, p = 0.037). Significant relative hypermethylation was seen at the gene level for RORC1 and RORC2 in SSc.
Zhou, et al. (39)	skin	The mRNA expressions of Th17 related cytokines IL-17A (p < 0.01), IL-21 (p < 0.001), IL-22 (p < 0.001), IL-17RA (p < 0.01), IL-21R (p < 0.001) and IL-22R1 (p < 0.05), Th1 related cytokine IFN-c (p < 0.01), Th2 related cytokine IL-4 (p < 0.01), Treg related cytokine TGF-β(p < 0.05) were significantly upregulated in SSc skin lesions in varying degrees compared with HC by RT-qPCR. Immunohistochemistry (IHC) analysis showed an over-expression of IL-17RA, IL-21R, and IL-22R1 and the Western blotting analysis showed an over-expression of IL-17A, IL-21, IL-21R, and IL-22R1 in early SSc skin lesions. The proportion of positive-stained cells of IL-17RA, IL-21R, and IL-22R1 in the epidermis of SSc and HC compared, the percentage of positive-stained cells of IL-17RA (p < 0.01), IL-21R (p < 0.001) and IL-22R1 (p < 0.05) increased in the lesions from the SSc compared with HC. The mRNA levels of IL-17A (r = 0.48, p < 0.05), IL-21 (r = 0.73, p < 0.001), IL-21R (r = 0.53, p < 0.05), IL-22 (r = 0.62, p < 0.01), IL-22R1 (r = 0.77, p < 0.001) and IL-26 (r = 0.50, p < 0.05) were correlated positively with the modified Rodnan skin score.
Kataoka, et al. (45)	blood	In FACS analysis, the proportion of Foxp3^+^ cells in total CD4^+^ T cells was decreased in patients with SSc compared with that in HC (p < 0.0001). The proportion of Foxp3^+^ cells in total CD4^+^ T cells was decreased in patients with late SSc compared with that in early SSc (p < 0.0036). Runx1 mRNA was reduced in CD25^+^ CD4^+^ T cells of patients with SSc compared to healthy subjects (relative expression: healthy subject, 1.14 0.43; SSc, 0.65 ± 0.31; p < 0.0015). Runx1 mRNA levels and proportions of Foxp3^+^ CD25^+^ cells in total CD4^+/^T cells of healthy subjects and patients with SSc (R^2^ = 0.375, p = 0.008).
Yang, et al. (40)	skin blood	By immunohistochemistry, the infiltration of IL-17^+^ cells, expressed as the number of cells showing superficial and deep dermal infiltration under × 400 magnification, was significantly increased in the skin from lesions of early SSc patients (superficial dermis: 7.5 ± 1.6 cells; deep dermis: 9.1 ± 1.8 cells) compared with late SSc patients (superficial dermis: 1.2 ± 0.8 cells; deep dermis: 1.0 ± 0.7 cells, P < 0.01) and healthy controls (superficial dermis: 0.8 ± 0.4 cells; deep dermis:0.6 ± 0.5 cells, P < 0.01). The number of the infiltration of Foxp3^+^ cells in the epidermis of lesional skin of early SSc patients (6.5 ± 1.2 cells) was significantly greater than that observed in the skin from late SSc patients was (2.2 ± 1.5 cells, P < 0.01) and healthy controls (1.0 ± 0.7 cells, P < 0.01). The infiltration of Foxp3^+^ cells in the superficial and deep dermis of early SSc patients (superficial dermis: 10.5 ± 1.6 cells; deep dermis:6.9 ± 1.7 cells) was significantly higher than that in patients with late SSc (superficial dermis: 2.2 ± 1.3 cells; deep dermis: 1.2 ± 0.8 cells, P < 0.01) and healthy controls (superficial dermis: 0.8 ± 0.4 cells; deep dermis: 0.8 ± 0.4 cells, P < 0.01). MTT assay showed that IL-17 from SSc patients promoted fibroblast growth. IL-17 induces collagen production in fibroblast, whereas IL-17 neutralizing antibody effectively blocked collagen production.
Papp, et al. (56)	blood	The significant negative correlation between the changes of the absolute numbers and percentages of peripheral CD95^+^ T and CD4^+^ CD25^+^ Treg cells (cell numbers, R = −0.533, p = 00.019; cell percentages, R = −0.459, p = 00.047) was observed. The negative correlations developed between the percentages of CD3^+^ CD69^+^ T cells (R = 0−0.528, p = 0.021) or CD3^+^ HLA-DR^+^ T cells (R = −0.464, p = 0.046) and the functional ability of CD4^+^ CD25^+^ Treg was observed after the last extracorporeal photochemotherapy cycle. The functional ability of Treg was assessed by suppression functional assay.
Mathian, et al. (57)	blood skin	In FACS analysis, Activated Tregs are decreased in all SSc patients irrespective of disease stage. Late SSc patients had signifi cantly less circulating resting Tregs than early SSc patients (0.60% (0.10 to 2.30) vs 1.02% (0.23 to 5.29) of CD4 T cells, respectively, p = 0.007). No differences in IL-17 serum levels were found between SSc patients at late and early disease stages (p = 0.57). The frequencies of Th17 cells did not significantly differ between early and late SSc patients (p = 0.5). A significantly increased proportion of late SSc patients (nine out of 13 patients) had circulating IL-22 levels above the detection threshold, as compared with early disease stage SSc patients (three out of 16 patients) (p=0.02). IL-22 transcripts were significantly more abundant in SSc skin, as compared with healthy skin samples (p=0.005).
Radstake, et al. (58)	blood	FACS analysis and Treg suppression assay were performed. Tregs obtained from SSc patients all had a markedly diminished suppressive capacity compared to those from healthy donors with T regs from late SSc, early dcSSc and late dcSSc suppressing CD4^+^ effector cell proliferation by, respectively, 28.2% ± 66.0 (p = 0.0001), 56.0% ± 68.5, (P = 0.006) and 18.3% ± 65.2 (p = 0.0001). TGF-β expression was significantly decreased by regulatory T cells obtained from SSc patients compared to those from healthy controls, and TGF-β expression on Tregs from late dcSSc and early dcSSc patients was significantly lower compared to that from patients with lcSSc (p = 0.008).
Radstake, et al. (59)	blood	In FACS analysis, the number of CD45Ro cells that co-expressed IL-17 was significantly increased in all SSc patients investigated (p = 0.0001), especially in early dcSSc compared with HC. Analyzing the serum cytokines, the levels of IL-6 (p = 0.0001), IL-1α (p = 0.002), and IL-23 (p = 0.003) were significantly higher in SSc as a whole compared with controls, although circulating IL-17 was not detectable.

SSc, systemic scleroris; dcSSc, diffuse cutaneous systemic sclerosis; lcSSc, limited cutaneous systemic sclerosis; FACS, fluorescence-activated cell sorting; RNA-seq, RNA-sequencing; SNP, Single Nucleotide Polymorphism; RT-qPCR, quantitative reverse transcription PCR.

Taken together, evidence has accumulated to indicate that CD4^+^ T cell subsets contribute to the pathogenesis of early SSc. In particular, Treg subsets have been considered in many past reports on early SSc in the context of the balance of other subsets, especially Th17 cells.

## Th17 Cells and Plasticity of Treg Cells

Th17 cells are characterized by secretion of IL-17A and their physiological role is the immunological defense against bacteria and yeast. In the context of the pathogenesis of SSc, IL-17A induces the production of pro-inflammatory cytokines, including chemokine (C-C motif) ligand 2 (CCL2), IL-6, and IL-8, by fibroblasts in human skin and lung (60, 61). IL-17A also affects vascular dysfunction through microangiopathy in SSc (60, 62, 63). IL-17A activates vascular smooth muscle cells *via* the ERK 1/2 pathway and increases collagen synthesis. Moreover, IL-17A promotes the migration of dermal vascular smooth cells. Therefore, Th17 cells could play variable roles in inflammation, fibrosis, and vascular dysfunction in SSc.

The heterogeneity of Th17 subsets is often discussed in view of the plasticity of Treg cells. It means that Treg cells have the capacity to convert into other Th cell subsets, including Th17 cells, in response to cytokine stimulation around their circumstances (64, 65). Recently, the plasticity of T cells was hypothesized as one of the causes of autoimmune diseases. For example, in collagen-induced arthritis mice, an animal model of rheumatoid arthritis (RA), CD25^lo^ Foxp3^+^ CD4^+^ T cells convert to Th17 cells, mediated by synovial fibroblast-derived IL-6 (66). These “exFoxp3 Th17 cells” accumulate in inflamed joints and have a strong ability to induce osteoclasts. IL-17^+^ Foxp3^+^ T cells are also detected in the synovium of people with active RA. As described in inflammatory arthritis, the plasticity of Tregs cells can be thought to contribute to the pathogenesis of many other autoimmune-mediated diseases.

## CD4 ^+^ Treg Cells

CD4^+^ Treg cells were first reported in 1995 as a cell population characterized by the surface expression of CD25 with immune suppressive function in mice (67). Thereafter, the transcription factor Forkhead box P3 (*FOXP3*) was reported as a critical master regulator for the differentiation and inhibitory ability of Treg cells (68). In humans, CD4^+^ CD25^+^ FOXP3^+^ Treg cells were also identified as an immune regulatory population (69). In addition, CD45RA expression defined the subpopulation of human Treg cells, that is, CD45RA-FOXP3^high^CD4^+^ Treg cells, as activated Treg cells with high regulatory activity (70). Transcriptomic analysis, especially single cell analysis, can determine detailed subpopulations of Treg cells. Zemmour et al. performed scRNA-seq of human and mouse Tregs and revealed the similarity of these species and the presence of subpopulations in each Treg subset (71).

Recently, the metabolic dynamism of Treg cells was unveiled. The conventional effector T cells including pathogenic Th17 have enhanced glycolysis, whereas Tregs are reliant on mitochondrial metabolism and oxidative phosphorylation rather than on glycolysis for energy production (72). In addition, oxidative phosphorylation in Tregs enforces Treg regulatory functions (73). On the other hand, under specific conditions, the metabolic status does not work to support Treg function. Shin et al. reported that oxidative phosphorylation facilitated TCR signaling and promoted Th17 cell-development, but not Treg development, under the existence of IL-6, TGF-β, and IL-23 (Th17 conditions) (74).

The association between the dysfunction of Treg cells and human immune-related diseases has been reported. Loss-of-function variants in the human *FOXP3* gene are known to cause IPEX (immune dysregulation, polyendocrinopathy, enteropathy, and X-linked) syndrome, which causes multiple autoimmune disorders due to Treg cell deficiency or dysfunction (75). Recently, anti-PD-1 and anti-CTLA-4 antibodies, which are immune checkpoint inhibitors, have been applied as therapeutic options for progressed malignancies. Treg cells suppress anti-tumor immunity in malignant tumors, and it is known that people with a large amount of Treg cell infiltration in the tumor region, such as people with gastric cancer and ovarian cancer, have a poor prognosis (76, 77). These antibodies promote anti-cancer immunological attack by suppression of Treg cell functions. Importantly, autoimmune diseases termed immune-related adverse events (irAEs) can develop because of treatment with immune checkpoint inhibitors. In addition, the important roles of Treg cells in suppressing the onset and progression of autoimmune diseases, for example, SLE, and fibrotic-related diseases have been reported (78, 79).

## Treg cells in the pathophysiology of SSc

Although it has not yet been thoroughly studied, the pathogenesis of SSc is based on immunological abnormalities as described above. The contribution of Treg cells to the pathophysiology of SSc has been explained by several mechanisms. First, the suppressive function of Treg cells in SSc patients is limited, causing the breakdown of the immune response, chronic inflammation, and fibrosis. Treg cells release some inhibitory cytokines, such as interleukin (IL)-10, TGF-β, and IL-35. These inhibitory cytokines function as immunosuppressive factors in various ways, such as suppressing the activation and maturation of immune cells (80). Several reports have pointed out the possibility of decreased inhibitory ability of Treg cells in SSc patients due to the decreased production of TGF-β and IL-10 (81). A second point is the promotion of fibrosis by pro-fibrotic cytokines produced by Treg cells. For example, TGF-β contributes to fibrotic pathology through the proliferation of fibroblasts. TGF-β promotes collagen production and extracellular matrix secretion, and also induces the epithelial-mesenchymal transition (EMT) (82). Some reports have suggested that Treg cells differentiate into Th2-like cells in SSc, and promote fibrosis through the production of cytokines, such as IL-4 and IL-13 (83). In this way, Treg cells are thought to be associated with several aspects of immune dysregulation and fibrosis during SSc pathogenesis.

In terms of TGF-β, there are three isoforms and TGF-β1 isoform is the major type. There remains unclear the respective roles of each TGF-β isotype in the pathogenesis of SSc. According to the studies reported so far, the sources of each TGF-β isotype are various including several immune cells and fibroblast/myofibroblast, and the role of each isotype is different depending on each cell type. For example, while many past reports referred to the relation of TGF-β1 and Treg or SSc (82), a recent report showed the epigenetic activation of *TGFB2* enhancer in the skin fibroblast from SSc (84). Of note, the TGF-β-measuring methods were different according to the past studies. That is, some reports showed the results of measured active TGF-β, and the others measured latent protein of TGF-β (82). Since TGF-β is secreted as the large latent complex, the results could differ depending on which types of proteins are targeted. That makes an issue complicated and must be organized.

In animal models of fibrosis and SSc, the pathophysiological roles of Treg cells have been investigated in detail. The topoisomerase-immunized mouse is one of the representative mouse models of SSc, and these SSc model mice showed increased production of IL-6, TGF-β1, and IL-17, and decreased production of IL-10 (85). In these mice, Th1, Th2, Th17, and Treg cells were increased in the bronchoalveolar lavage fluid of the fibrotic lungs. In the other model of SSc, mice hetero-deficient in the transcription factor Friend leukemia virus integration 1 (*Fli1*), it has been reported that the induction rate of Treg cells in the skin at 1 week after the completion of bleomycin administration is lower than that in wild-type mice, which may contribute to the exacerbation of skin fibrosis (86). There were also several reports describing Treg cells in the classical model of fibrosis, the bleomycin-induced pulmonary fibrosis model. Birjandi et al. reported that an increase of CD4^+^CD25^high^FoxP3^+^ Treg cells in the lung resulted in a shift to a type 2 immune response and the exacerbation of lung fibrosis (87). On the other hand, Kamio et al. reported that lung fibrosis was reduced by Treg cell transfer 14 days after bleomycin administration (88). In addition, Boveda-Ruiz et al. demonstrated that the prognosis improved in a bleomycin-induced pulmonary fibrosis model when Treg cells were removed in an early phase by anti-CD25 antibody administration. They also reported that lung fibrosis was exacerbated when Treg cells were removed in a late phase of the disease. These reports suggested that the pathological roles of Treg cells in lung fibrosis may differ depending on the stage of disease (89).

## Imbalance of Treg Cells and Th17 Cells in SSc

As mentioned in the section of “Th17 cells and plasticity of Treg cells”, the plasticity of Treg cells and the imbalance of Treg cells and other subsets, especially Th17 cells, have been focused on in the pathogenesis of SSc (90). For example, Th17 cells produce the inflammatory cytokine IL-17A, which promotes the proliferation and migration of dermal vascular smooth muscle cells and is involved in the vasculopathy of SSc (91). It was also suggested that IL-17A contributes to the transformation of myofibroblasts and promotes skin and lung fibrosis (92). In the case of SSc, several reports proposed that some Treg cell populations were transformed into Th17 or Th17-like cells in SSc. Liu et al. reported that CD4^+^ CD25^+^ FOXP3^low^CD45RA-T cells were increased in the peripheral blood of people with SSc and that this population of cells produced high levels of IL-17, like Th17 cells (93). In our previous report on FACS analysis of peripheral blood, the frequency of Th17 cells was increased in the early phase of SSc patients compared to the late phase of SSc and HC (42). According to the reports on the skin lesions in SSc, Yang et al. performed immunohistochemistry using skin samples from people with active SSc and revealed the increased infiltration of IL-17^+^ cells in the skin (40). Based on FACS analysis, they also reported that elevated percentages of circulating Th17 cells and Th17-derived IL-17A were involved in fibroblast growth and collagen production in SSc. Truchetet, et al. also showed that the numbers of IL-17A producing cells correlated with the expression of CCR6 and indicated that these cells were prone to be recruited into the inflamed target tissue, for example, the skin and the lung in SSc (94). 

The imbalance between Treg and Th17 cells could also contribute to gastrointestinal disorders in SSc. Gastrointestinal disorders are a very frequent problem in SSc patients (95). Any area of the gastrointestinal tract can be damaged, and the esophagus is particularly vulnerable (96). Nicola et al. investigated the concentrations of Treg, Th17, and Th1-related cytokines in the digestive juices of SSc patients (97). They showed higher concentrations of IL-17A and lower concentrations of IL-10 and TGF-β in SSc patients with esophageal involvement. Interestingly, IL-17A concentrations were increased in minor inflammatory gastritis compared to those in advanced atrophic gastritis. These results suggested that the imbalance between Treg and Th17 cells contributes to the early phase of gastrointestinal disorders in SSc. The authors also suggested that the overproduction of IL-17A contributed to gastrointestinal fibrosis, especially in the early stage of the disease. Thus, Th17 cells are a major player in the pathogenesis of SSc, and the imbalance between Treg and Th17 cells could promote the pathogenic course of gastrointestinal diseases.

In the context of IL-17 and fibrosis, the results were still controversial. Some past reports showed that the modified Rodman skin thickness score (MRSS) tended to be lower in patients with elevated IL-17A than those without (98, 99). Truchetet, et al. reported that the numbers of IL-17A^+^ cells were increased in SSc, and they inversely correlated with the MRSS (100). In addition, Nakashima, et al. reported that IL-17A signaling had an antifibrogenic effect *via* the upregulation of miR-129-5p and the downregulation of connective tissue growth factor and α1(I) collagen (99). Some other reports also showed the anti-fibrotic effects of Th17 or IL-17A (100–103).

Even though, considering the imbalance of Treg and Th17 cells could be one of the major pathogenic processes in SSc, targeting Th17 cells and/or IL-17 will be a promising treatment strategy for SSc. In terms of the mice models of SSc, Okamoto, et al. found that IL-17A deficiency reduced skin fibrosis in the bleomycin-induced mice and the TSK-1 model (104). Park, et al. also reported on the efficacy of targeting IL-17 in bleomycin-induced mice and chronic graft-versus-host disease models (105). Using Secukinumab, a monoclonal antibody against IL-17A, Karatas, et al. showed the amelioration of bleomycin-induced dermal fibrosis in mice (106). In humans, an active but not recruiting phase III trial of brodalumab (AMG 827), a human, anti-IL17 receptor monoclonal antibody, for SSc (Clinicaltrials.gov identifier: *NCT03957681*) is undergoing. Although further data accumulation is needed, correction of the imbalance of Treg and Th17 cells could be a promising therapeutic strategy for SSc.

## Treg Cells in Early SSc Patients

There have been many reports about the frequencies and absolute numbers of Treg cells in the peripheral blood and tissues of SSc patients. However, the results were inconsistent between the studies. For example, using immunostaining, Antiga et al. determined that FOXP3^+^ Treg cells were less infiltrated in SSc skin tissues than HC (107). At the same time, they reported that serum TGF-β and IL-10 concentrations and the frequencies of peripheral blood CD4^+^ CD25^bright^FOXP3^+^ cells were decreased in SSc patients (107). In contrast, Yang et al. reported that the numbers of FOXP3^+^ Treg cells increased in the skin tissue of people in the early phase of SSc compared to people in the late phase of SSc and HC. The authors proposed that this increase of Treg cells suggested a regulatory feedback reaction for the exacerbation of immune response in the onset of SSc (40). The differences in the definitions of Treg cells, such as the surface markers, could cause these controversial results. Additionally, some populations of CD4^+^ CD25^+^ cells secret pro-inflammatory cytokines, which could cause the inconsistent results of the previous reports about the frequencies and functions of Treg cells in SSc. Different backgrounds of SSc patients in the studies could also contribute to the variation in the results. In recent years, a meta-analysis of peripheral blood Treg cell/CD4^+^ T cell ratios was reported by Deng et al. They reported that there was no statistically significant difference between the SSc patients and HC (108). However, in view of a variety of stages and clinical phenotypes in SSc, such a meta-analysis would not lead to a meaningful result. Indeed, Frants et al. reported that Treg cells tended to be higher in people in the early phase of SSc (81), and the frequencies of Treg cells would depend on the phase of the disease. In recent studies, some reports also showed the increase of Treg subsets or Treg subpopulations in early SSc (42–44, 58), although some studies reported the decrease of Treg subsets in early SSc (41, 45).

In addition, many reports suggested the functional inability of peripheral blood Treg cells to suppress the immune response in early SSc. Ugor et al. reported that the production of immunosuppressive cytokines by Tregs was diminished in early SSc (43). Radstake et al. showed a lower immunosuppressive ability of SSc patient-derived Treg cells in both early and late SSc (58). In this way, the frequencies and functions of Treg cells in SSc remain controversial. As they would depend on the phases and clinical phenotypes of SSc, these points should be considered in understanding the roles of Treg cells in SSc.

Our team recently published a catalogue of gene expression in the 28 immune cells from people with 10 autoimmune diseases, including SSc, called ImmuNexUT (Immune Cell Gene Expression Atlas from the University of Tokyo) (109). In this study, a cross-sectional analysis of multiple autoimmune disorders revealed that the expression of IFN-signature genes was high in autoimmune diseases such as SLE, and that IL-18 or IL-1β signature was high in autoinflammatory diseases such as Behcet’s disease. Notably, the gene expression of SSc patients varied greatly among individuals. Some showed a transcriptome similar to an autoimmune disease, while others had a transcriptome that similar to an autoinflammatory disease. These results suggested that SSc patients were immunologically heterogeneous, and this heterogeneity may cause the difficulty of obtaining consistent results in SSc.

## Transcriptome Analysis of Treg Cells in Early SSc Patients

As described above, the clinical features differ between the early and the late stages of SSc (1). In the early phase of SSc, inflammation and autoimmune responses were prominent (14), whereas pro-fibrotic reactions were evident in the late phase of the disease. Therefore, the analysis of SSc-related immunological data for each stage of SSc could unveil stage-specific immunological disturbances. Therefore, our team performed FACS and RNA-seq analysis of peripheral blood immune cells in early SSc (defined as morbidity of less than 5 years) and late SSc (defined as morbidity of 5 years or more) to investigate the immunological characteristics of early SSc patients. As a result, in the comparison of early SSc vs. late SSc patients, the frequencies of Th17 cells were significantly higher in early SSc. In contrast, no significant difference was observed in the frequencies of the fraction II effector regulatory T cells (Fr. II eTreg) subset defined by CD4^+^CD25^++^ CD45RA- in FACS-based data. Next, we estimated the proportion of Treg subsets and subpopulations by deconvolution based on the previously reported single cell RNA-seq data. Interestingly, among the transcriptome of 24 cell subsets, differences in gene expression in Treg cells were only evident between early and late SSc. In addition, the proportion of “activated subpopulations” of Treg cells was significantly higher in early SSc, in association with a higher expression of activation markers. Pathway analysis suggested that oxidative phosphorylation was enhanced in “activated subpopulations” of Treg cells in early SSc. Treg cells rely on the oxidative phosphorylation pathway for most of their energy production (72). This result suggested that regulatory feedback for the activation of the immune system occurs in the early stage of SSc pathogenesis, or alternatively, a compensative feedback for the decrease in the immunosuppressive ability of Treg cells as reported previously (81). It was also reported that enhancement of the oxidative phosphorylation pathway promotes Th17 differentiation, and inhibition of the oxidative phosphorylation pathway induces differentiation into Treg cells (74). The enhanced oxidative phosphorylation pathway in early SSc Treg cells would represent a metabolic sign of transition of Treg to Th17 cells. In this study, we also performed differentially expressed gene (DEG) analysis and identified hub genes that regulate the expression of DEGs between early and late SSc in the gene network estimated with a non-knowledge-based method. This data-driven approach identified *ARPC3* as a hub gene. *ARPC3* is a crucial gene for immune synapse formation and T-cell receptor (TCR) signaling (110, 111). This result implied that the different gene expression patterns of Tregs between early SSc and late SSc were regulated by TCR signaling. This finding was consistent with previous reports suggesting that TCR signaling was essential in Tregs and was required for suppressive activity and differentiation to an activated phenotype (112, 113). In this study, the expression of *ARPC3* was upregulated in early SSc. Therefore, it could enhance the sensitivity of TCR signaling and induce activation of Fr. II eTregs.

In previous reports, Treg cells in the peripheral blood of SSc patients were contradictory in both quantitative and qualitative evaluations. Our study focused on the subpopulation of activated Treg cells in early SSc patients. Therefore, we propose that meaningful results could be obtained by analyzing a “subpopulation” of Treg cells derived from homogeneous phases of disease and clinical features of SSc patients.

## Discussion

**Figure 1:**
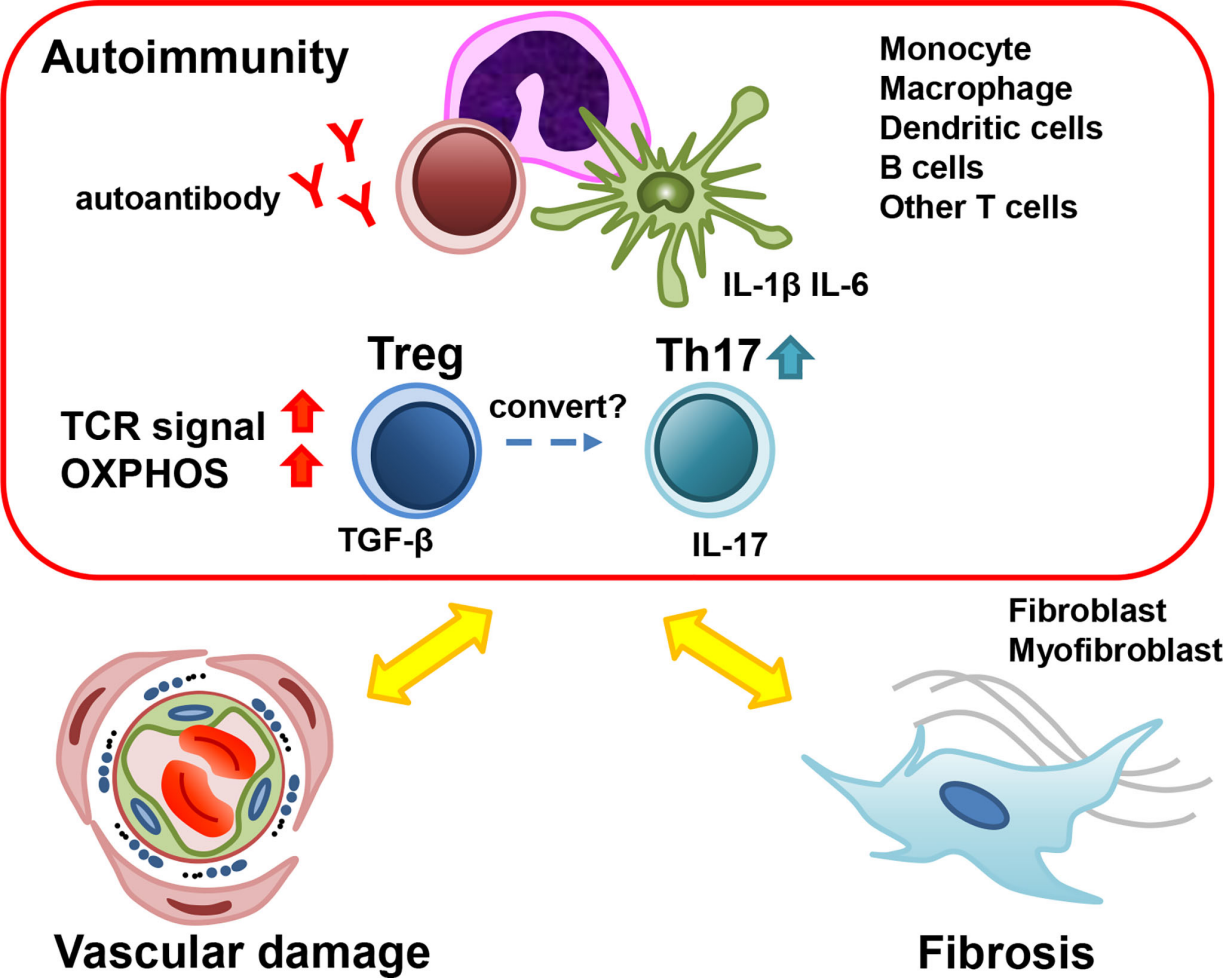
The role of Treg in the pathogenesis of early SSc. Many types of cells contribute to the pathogenesis of early SSc. Treg cells secrete TGF-β, which promotes myofibroblast activation and fibrosis. The dysfunction of Treg cells results in the development of autoimmunity and inflammation. Under the inflammatory condition, Treg could convert into Th17 and Th17 could promote inflammation and vasculopathy. SSc; systemic sclerosis, Treg; regulatory T cells, Th17; T-helper17 cells, TGF-β; transforming growth factor-β.

We outlined the current understanding of the roles of Treg cells in SSc. It was suggested that Treg cells contribute to the pathophysiology of SSc *via* the decreased inhibitory ability and disruption of equilibrium with other helper T cell subsets (**Figure 1**). Here, our team demonstrated an increase in the Treg “activated subpopulation” accompanying an increase in oxidative phosphorylation in early SSc patients (42). In addition, an increase in Th17 cells was also observed in early SSc patients. These results suggested that the imbalance between Treg and Th17 plays an important role in the pathophysiology of early SSc. Importantly, some reports showed that targeting IL-17 was effective to improve the skin and lung fibrosis in SSc models (104–106). Even though it is still controversial and further studies will be needed (99), this imbalance between Treg and Th17 cells could be a therapeutic target of SSc, and control of abnormal Treg activation could be another therapeutic strategy for SSc. From the point of view of Treg plasticity, it could be effective to ameliorate inflammation using anti-cytokine agents, because the conversion of Treg into Th17 is prompted under the specific inflammatory condition (64). It was reported that tocilizumab, an anti-IL-6 receptor monoclonal antibody, was partially effective to preserve the lung function in SSc with interstitial lung diseases in the early disease phase (114). We suspected that tocilizumab could control the inflammatory environment and prevent Treg to Th17 conversion. Further studies of Treg cells in SSc will provide additional clues to solve the problem. Our study suggests that a detailed definition of Treg cell populations and a homogeneous stage and pathological background of the study target groups will be required to attain high quality and reliability of the results in SSc studies.

## Author Contributions

SK, YN, HS, and KF designed the study and contributed to writing the manuscript. HS and KF supervised the study. All authors were involved in drafting the article or revising it critically for important intellectual content, and all authors approved the final version to be published.

## Conflict of Interest

YN belongs to the Social Cooperation Program, Department of Functional Genomics and Immunological Diseases, supported by Chugai Pharmaceutical.

The remaining authors declare that the research was conducted in the absence of any commercial or financial relationships that could be construed as a potential conflict of interest.

## Publisher’s Note

All claims expressed in this article are solely those of the authors and do not necessarily represent those of their affiliated organizations, or those of the publisher, the editors and the reviewers. Any product that may be evaluated in this article, or claim that may be made by its manufacturer, is not guaranteed or endorsed by the publisher.
